# Exploratory study on a novel automatic quantification software by artificial intelligence for geographic atrophy associated to age-related macular degeneration

**DOI:** 10.1186/s40942-026-00862-x

**Published:** 2026-04-30

**Authors:** Jorge Ruiz-Medrano, Aiwen Su, Zaixing Mao, Masahiro Akiba, José M. Ruiz-Moreno

**Affiliations:** 1 Puerta de Hierro-Majadahonda University Hospital, C/Manuel de Falla 1, Madrid, 28222 Spain; 2 Department of Ophthalmology, Universidad Autónoma de Madrid, Madrid, Spain; 3 Topcon Corporation, Tokyo, Japan; 4 Department of Ophthalmology, Castilla La Mancha University, Albacete, Spain

**Keywords:** Geographic atrophy, Artificial intelligence, Age-related macular degeneration, Automatic, GA, AMD, AI

## Abstract

**Objectives:**

To determine the accuracy and repeatability of novel artificial intelligence (AI)–based software for automated quantification of geographic atrophy (GA) associated with age-related macular degeneration (AMD) using swept-source optical coherence tomography (SS-OCT) images.

**Methods:**

This cross-sectional observational study included 33 eyes from 33 patients with GA secondary to AMD and one healthy control eye, all without a history of choroidal neovascularization. Each eye underwent three consecutive 7 × 7 mm^2^ SS-OCT scans (Topcon Corporation, Tokyo, Japan). Two independent observers manually delineated GA areas on en face OCT images in a masked fashion at two time points separated by 15 days. Individual and mean manual measurements were compared with automated AI-derived GA measurements, yielding a total of 165 measurements. Lesions were categorized according to morphology (regular vs. irregular) and number (single vs. multiple). Agreement between manual and AI measurements was assessed using Lin’s concordance correlation coefficient (CCC). Software repeatability was evaluated using the intraclass correlation coefficient (ICC) based on a two-way mixed-effects model.

**Results:**

The mean patient age was 73.4 ± 11.7 years. Twelve eyes exhibited regular lesion morphology, and 11 eyes had single GA lesions. Intra-observer repeatability was excellent, with ICCs of 0.999 (95% CI, 0.997–0.999) for Observer X and 0.999 (95% CI, 0.999–0.999) for Observer Y. Agreement between the mean manual measurements of both observers was also high (ICC = 0.997; 95% CI, 0.989–0.999). Agreement between AI-based measurements and the mean of the four manual annotations was strong for the overall cohort (CCC = 0.992; 95% CI, 0.981–0.998). Stratified analyses showed CCCs of 0.989 (95% CI, 0.816–0.997) for regular lesions and 0.988 (95% CI, 0.944–0.998) for irregular lesions, and 0.999 (95% CI, 0.998–1.000) for single lesions and 0.984 (95% CI, 0.963–0.995) for multiple lesions. Agreement by lesion size was lower for small lesions (< 3.7 mm^2^; *n* = 12; CCC = 0.903; 95% CI, 0.742–0.974) and higher for medium (3.7–9.4 mm^2^; *n* = 13; CCC = 0.978; 95% CI, 0.940–0.994) and large lesions (> 9.4 mm^2^; *n* = 8; CCC = 0.956; 95% CI, 0.676–0.998).

**Conclusions:**

This AI-based software demonstrates high accuracy and excellent repeatability for automated GA area quantification across different lesion morphologies, numbers, and sizes, with minimal processing time. These findings could support its potential utility in routine clinical practice and research applications.

**Clinical trial number:**

Not applicable.

## Introduction

Geographic atrophy (GA) represents the advanced stage of non-exudative age-related macular degeneration (AMD) and has recently attracted increased clinical and research interest. The approval by the U.S. Food and Drug Administration (FDA) of two pharmacological agents that slow GA progression has positioned GA area measurement as a key outcome variable in both clinical practice and clinical trials.

Historically, GA has been defined using color fundus photography (CFP) as a well-demarcated area of retinal pigment epithelium (RPE) loss with visible underlying choroidal vessels and a minimum diameter of 175 μm [1]. However, fundus autofluorescence (FAF) imaging using blue light at 488 nm has largely replaced CFP as the gold-standard modality for GA assessment, owing to its superior contrast in delineating lesion borders [2]. FAF imaging is based on the detection of autofluorescence primarily derived from lipofuscin, a byproduct of the visual cycle that accumulates within RPE cells. On FAF, GA manifests as one or more regions of markedly reduced or absent autofluorescence signal corresponding to RPE cell loss, appearing as sharply demarcated hypoautofluorescent (black) areas. Consequently, FAF has become the standard imaging modality for evaluating GA progression by measuring lesion growth at the RPE level [3]. GA area growth rate has been widely adopted as a primary endpoint in interventional clinical trials and is recognized by the FDA for regulatory approval [4].

Despite its widespread use, FAF does not allow direct assessment of photoreceptor (PR) integrity [5]. Furthermore, FAF image quality may be compromised by factors such as small pupil size or media opacities, which are common in the GA patient population. In contrast, optical coherence tomography (OCT) has provided substantial insights into the pathomorphological features of GA, enabling in-depth visualization of retinal layer alterations [6]. In recent years, multiple studies have explored automated approaches for GA detection and quantification using OCT-based imaging [7–14].

The aim of this study is to evaluate the accuracy and repeatability of novel artificial intelligence (AI)–based software designed to automatically quantify GA areas associated with AMD using swept-source optical coherence tomography (SS-OCT) images.

## Subjects and methods

This cross-sectional observational study was approved by the Ethics Committee of Puerta de Hierro University Hospital (approval number 01/25) and conducted in accordance with the Declaration of Helsinki, International Council for Harmonisation (ICH) guidelines, Good Clinical Practice (GCP) standards, and applicable Spanish regulations. Patient confidentiality was ensured by encrypting or anonymizing all identifying information. Every participant in the study signed the appropriate informed consent.

A total of 33 images of geographic atrophy (GA) lesions from 33 different patients were acquired using a swept-source optical coherence tomography (SS-OCT) device (DRI OCT Triton; Topcon Corporation, Tokyo, Japan). Scans were obtained in 3D cube mode using a 7 × 7 mm protocol centered on the macula. For each volumetric dataset, an en face image was generated by summing signal intensity across all depth layers, yielding a projection view of the retinal structure. Eyes with previous or active macular neovascularization (MNV), as identified on OCT, were excluded. In addition, eyes with peripapillary atrophy in contact with GA, as well as any other lesions that could interfere with accurate quantification, were also excluded. According to Ray et al., only one eye per participant was included in the analysis [15].

The inclusion criteria for OCT 3D volume scans were as follows: absence of blink artifacts, eye motion artifacts, and defocus, as well as an image quality score greater than 40 as provided by the device. All images included in this study satisfied these criteria.

**Fig. 1:**
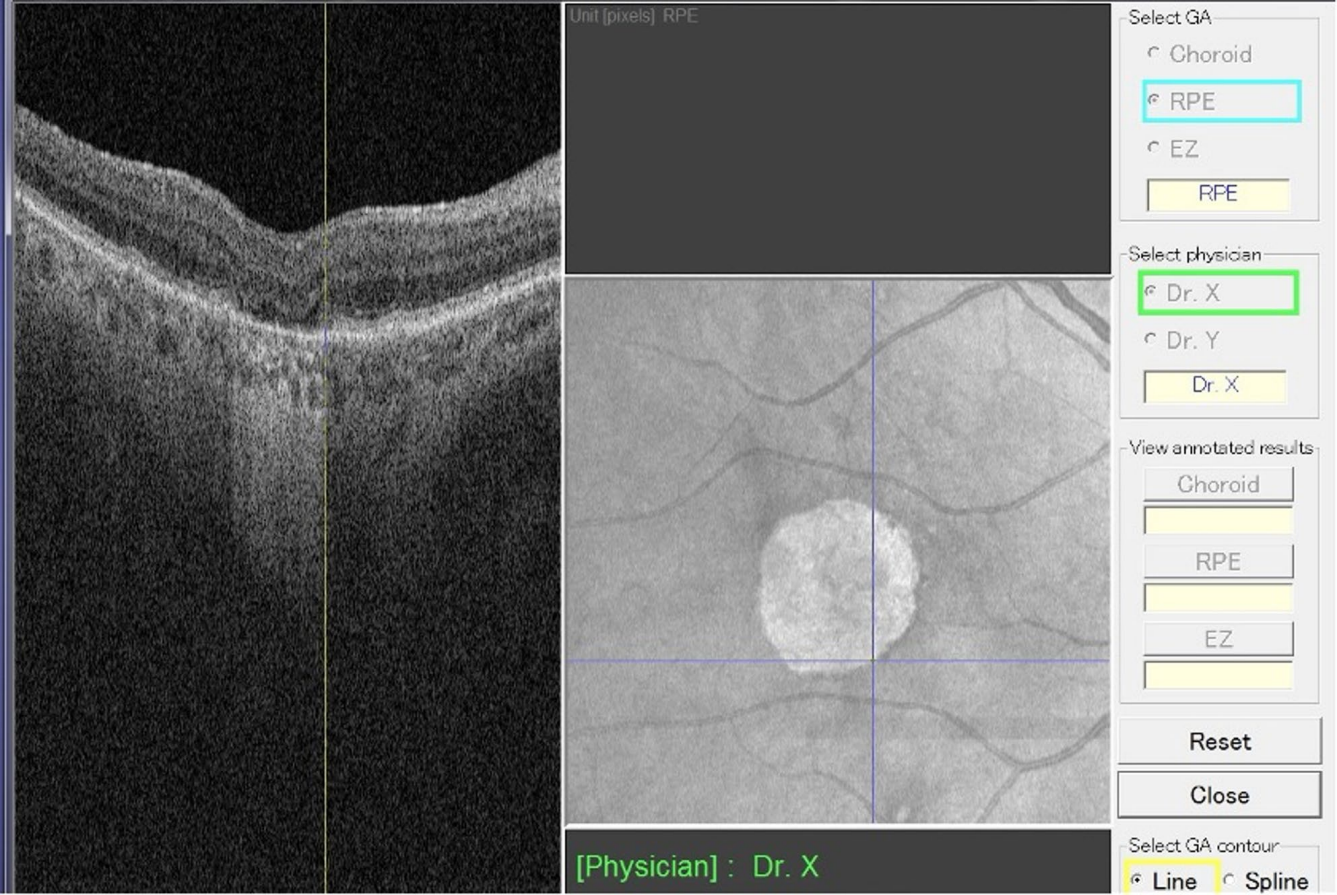
Screenshot of the manual GA software tool used during the development of this study

Each *en face* image was independently annotated by two ophthalmologists (Observer X and Observer Y), who manually delineated the GA lesion boundaries on two separate occasions (Fig. 1). Data were exported to dedicated software for analysis. The observers delineated GA regions on *en face* images while simultaneously referencing the corresponding B-scan images on a point-by-point basis. Specifically, areas of retinal pigment epithelium (RPE) disruption were identified on the B-scans and used to guide the corresponding annotations on the *en face* images.

**Fig. 2:**
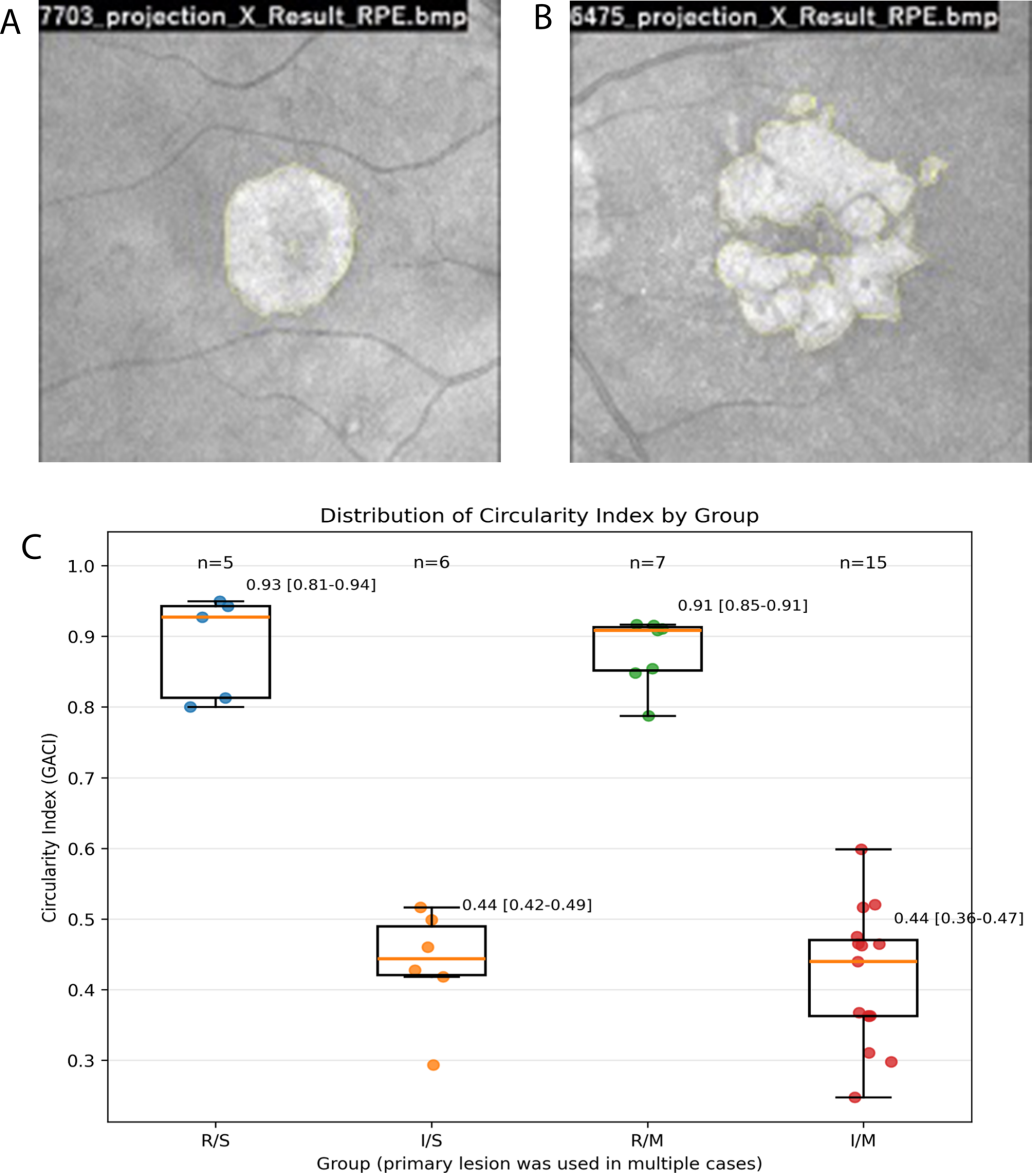
Clinical examples of age-related macular degeneration geogeraphic atrophy lesions: Regular (**a**), irregular (**b**). (**c**) Distribution of circularity index by group

The annotation sessions were conducted two weeks apart to minimize recall bias, resulting in four annotation masks per image. Additionally, all lesions were manually classified according to morphology (regular vs. irregular, Fig. 2a and b) and lesion number (single vs. multiple). For classification into regular versus irregular morphology, the circularity index [16] was calculated for each lesion in each eye. Lesions were classified as regular when the circularity index exceeded 0.6, either in the case of a single lesion or in the largest lesion in eyes with multiple lesions. This approach was adopted because, if all lesions within eyes with multiple lesions were included in the calculation, such cases would invariably be classified as irregular. The aim of this classification was to assess lesion morphology rather than to evaluate risk of progression (Fig. 2c; Table 1).

**Table 1:** . Lesion characteristics

Case	Eye	Area	Perimeter	Primary Area	Primary Perimeter	GACI Primary	GACI	*R*/I	S/M
1	R	32754,00	2427,17	9833	515,82	0,4643	0,0698	I	M
2	R	56001,00	2653,72	45,734	1389,03	0,2978	0,0999	I	M
3	L	37460,00	1953,63	17,810	785,26	0,3629	0,1233	I	M
4	R	2367,00	474,90	834	148,50	0,4752	0,1318	I	M
5	L	33350,00	1755,74	17,323	937,10	0,2478	0,1359	I	M
6	R	39530,00	1875,27	23,874	802,85	0,4654	0,1412	I	M
7	R	14135,00	1082,63	5104	264,67	0,9155	0,1515	R	M
8	R	10319,00	881,44	6169	302,33	0,8481	0,1669	R	M
9	L	14748,00	1020,87	11,231	566,60	0,4396	0,1778	I	M
10	R	8028,00	701,58	4990	282,19	0,7874	0,2049	R	M
11	R	42112,00	1565,79	38,518	1248,14	0,3107	0,2158	I	M
12	L	26619,00	1205,33	20,534	706,90	0,5163	0,2302	I	M
13	L	40678,00	1461,82	35,751	1112,50	0,3629	0,2392	I	M
14	L	48802,00	1512,47	47,641	1285,15	0,3624	0,2680	I	M
15	R	30492,00	1143,07				0,2932	I	S
16	R	49239,00	1396,61	48,428	1287,14	0,3673	0,3172	I	M
17	R	66757,00	1552,90	64,522	1163,45	0,5989	0,3478	I	M
18	R	3840,00	367,69	2871	199,29	0,9083	0,3569	R	M
19	L	4248,00	384,29	3338	221,54	0,8546	0,3614	R	M
20	R	44802,00	1211,43	43,933	1092,35	0,4626	0,3836	I	M
21	R	91704,00	1660,21				0,4180	I	S
22	R	55145,00	1273,29				0,4274	I	S
23	L	53965,00	1240,28	53,145	1132,81	0,5204	0,4408	I	M
24	R	14220,00	636,33	7908	330,35	0,9105	0,4413	R	M
25	R	47820,00	1142,57				0,4603	I	S
26	R	40809,00	1013,64				0,4991	I	S
27	L	62099,00	1228,98				0,5166	I	S
28	L	26665,00	681,90	26,227	599,60	0,9166	0,7206	R	M
29	L	8568,00	366,80				0,8002	R	S
30	L	73757,00	1067,70				0,8130	R	S
31	R	18840,00	505,23				0,9274	R	S
32	L	3138,00	204,52				0,9426	R	S
33	R	17984,00	487,77				0,9498	R	S

R: Right; L: Left; GACI: Geographic Atrophy Circularity Index; R: Regular; I: Irregular; S: Simple; M: Multiple

### AI model architecture and training

**Fig. 3:**
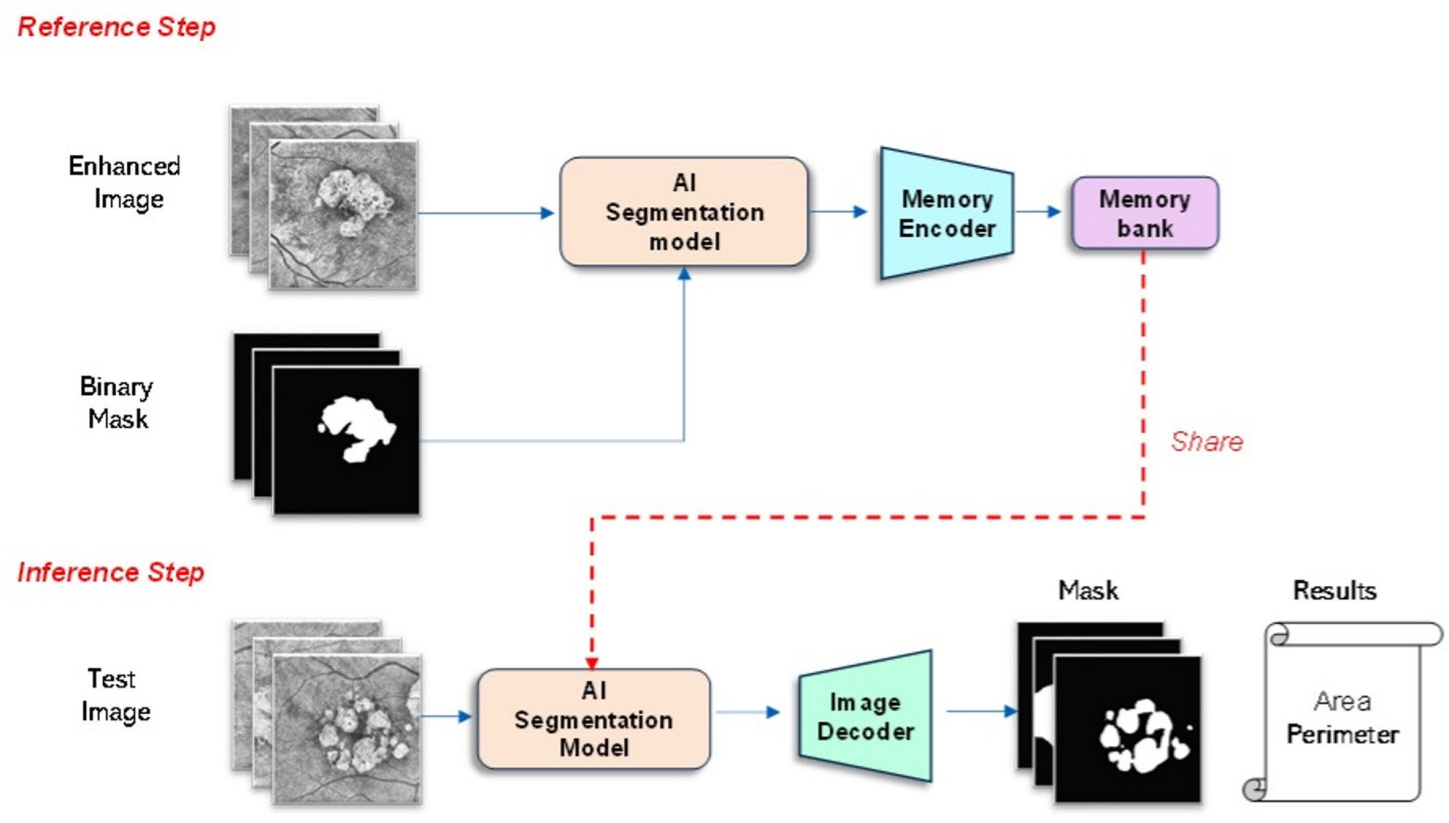
Overall workflow of SAM2 with memory prompting for GA segmentation. We adopt SAM2 for GA segmentation, leveraging its two-stage process: memory construction from annotated reference samples, and guided inference on new inputs. (**a**) During the reference phase, annotated GA images are used to extract features and build the memory bank that storing object-specific embeddings. (**b**) During the inference phase, the input image is encoded and interacts with the memory bank, guiding the decoder to predict segmentation masks

The artificial intelligence (AI) system employed a two-stage framework consisting of a reference (memory construction) stage and a memory-guided inference stage (Fig. 3). Of the 33 images, 14 were assigned to the reference dataset and 19 to the test dataset.

During the reference stage, 14 en face reference images and their corresponding binary annotation masks were processed using the SAM2 model to extract dense feature representations, which were stored in a memory bank. Eight distinct models were constructed using annotations from different observers and lesion subtypes, thereby encoding observer-specific annotation patterns and lesion-specific characteristics.

For inference, the same SAM2 model was applied to extract features from a query image. These features were fused with the corresponding memory bank and passed to the decoder to generate a per-pixel probability map, where values p ∈ [0, 1] represented the likelihood of each pixel belonging to a GA lesion. The probability map was binarized using a threshold of 0.5 to produce the final segmentation mask. Lesion area and perimeter were subsequently calculated based on the number of pixels within the segmented region and along its boundary, respectively.

### Statistical analysis

Agreement between AI-derived measurements and manual annotations was assessed using Lin’s concordance correlation coefficient (CCC). The repeatability of the software-derived measurements was evaluated using the intraclass correlation coefficient (ICC) based on a two-way mixed-effects model. Intra-observer consistency was assessed by comparing annotations performed by the same observer at the two time points, and mean measurements between observers were also compared. Bland–Altman plots were generated to visually assess agreement and to identify systematic bias between predicted and manually annotated GA lesion areas.

Data analysis was performed using Python 3.10. Statistical significance was set at *P* < 0.05.

## Results

The mean patient age was 73.4 ± 11.7 years. 16 cases exhibited regular lesion morphology, 17 cases showed irregular lesions; 11 eyes presented with a single GA lesion and 22 showed multiple lesions.

**Fig. 4:**
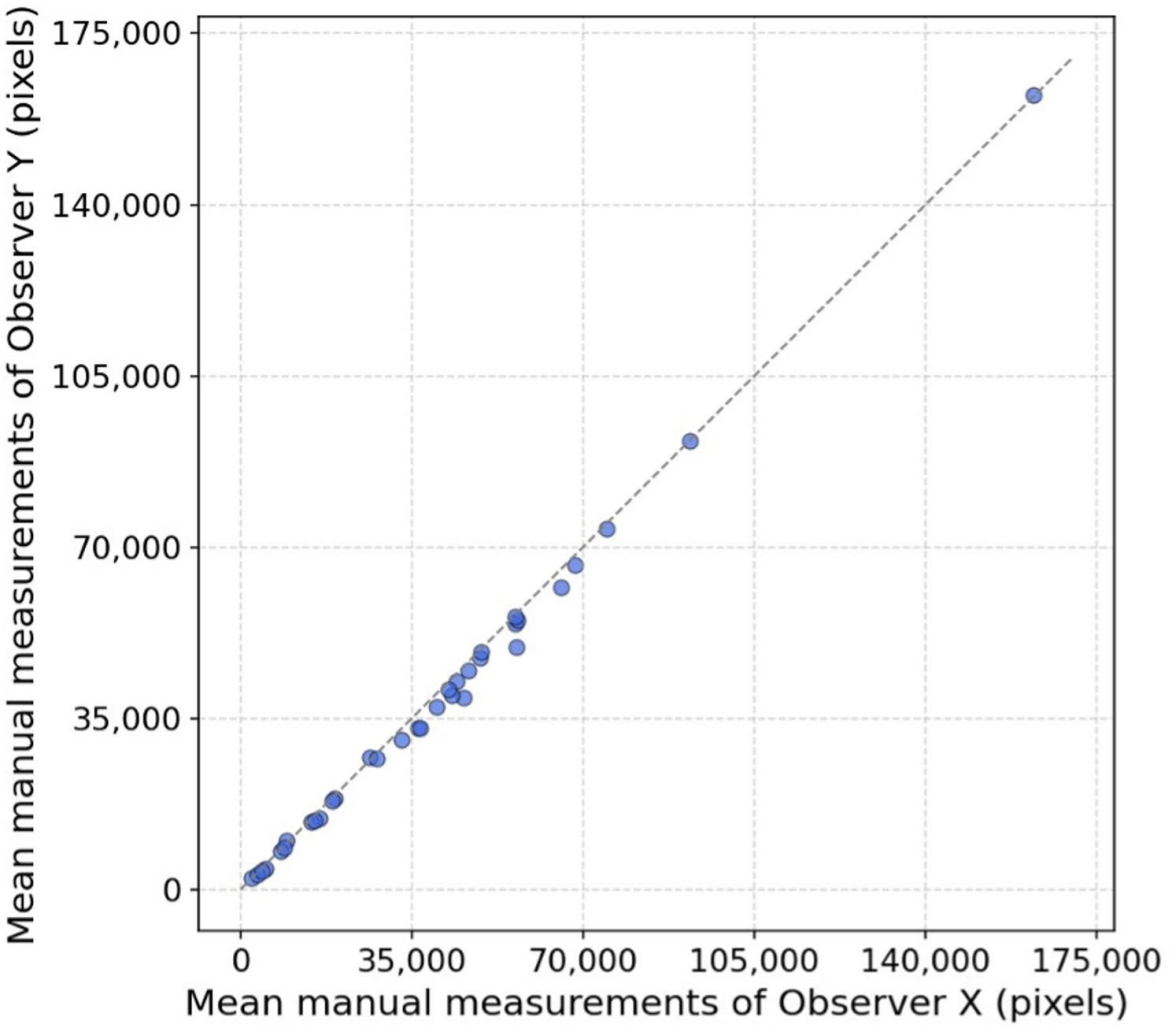
Scatter plot comparing the mean of the two measurements for each investigator

Intra-observer reproducibility was excellent for both graders. The intraclass correlation coefficient (ICC) for repeated measurements by Observer X was 0.999 (95% CI, 0.997–0.999), and for Observer Y was 0.999 (95% CI, 0.999–0.999). Agreement between the mean manual measurements of Observers X and Y was similarly high, with an ICC of 0.997 (95% CI, 0.989–0.999) (Fig. 4).

**Fig. 5:**
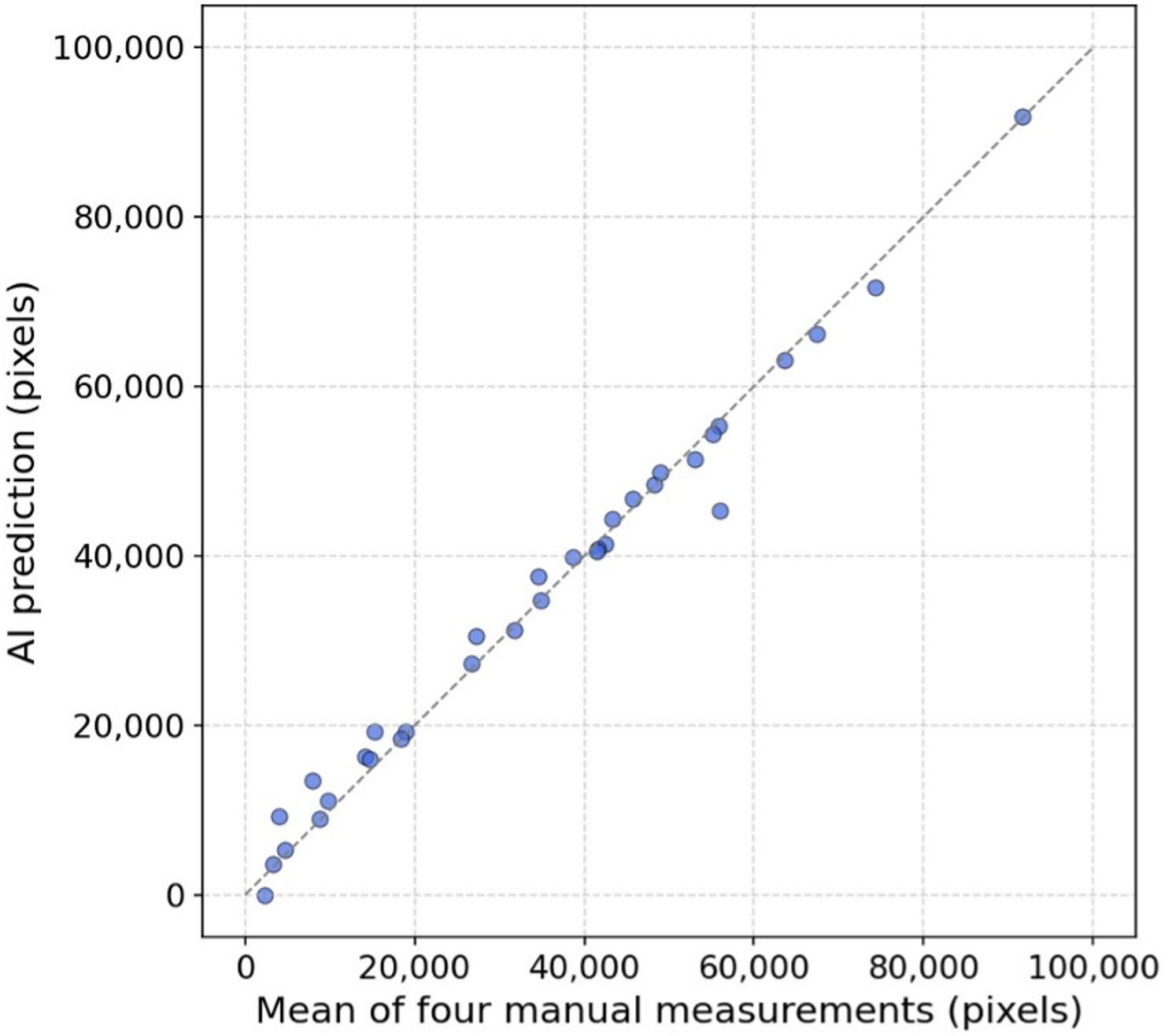
Scatter plot comparing the mean of the four measurements from the investigators versus artificial intelligence

**Fig. 6:**
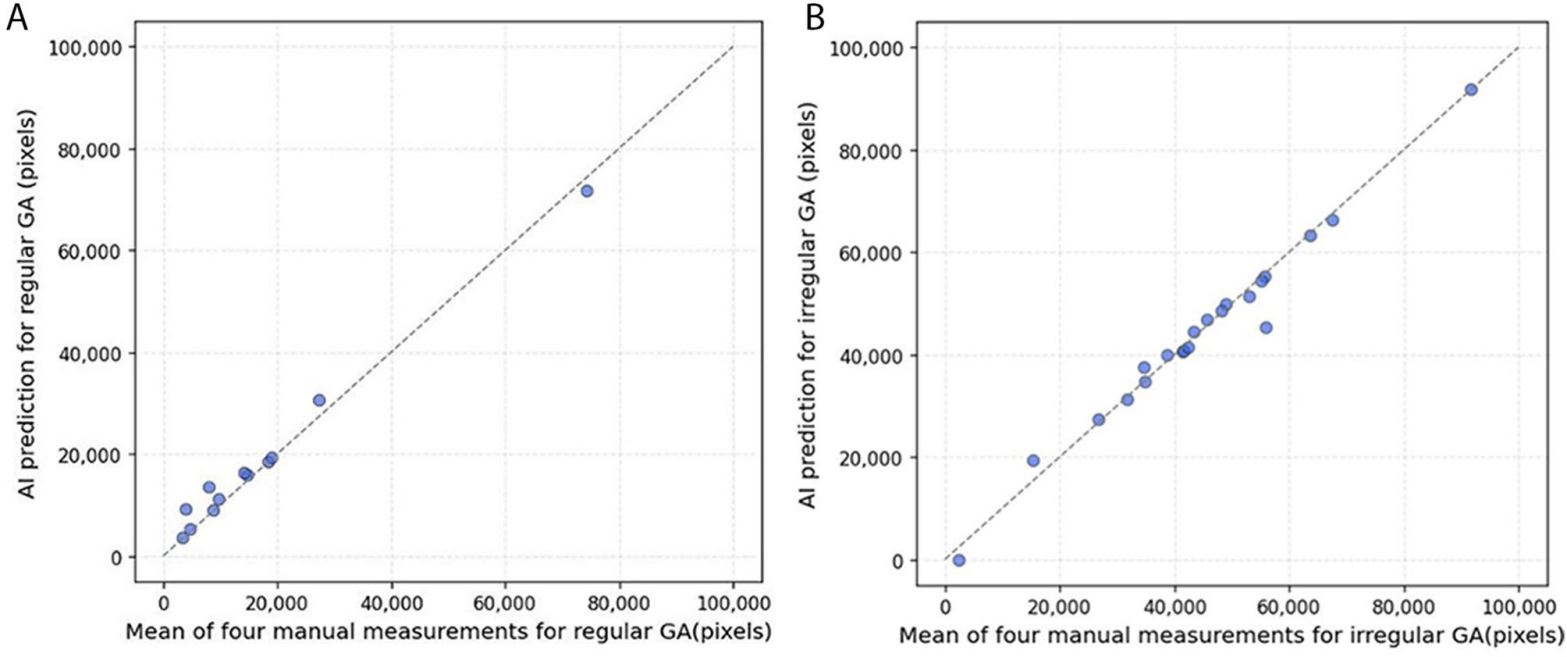
Scatter plot comparing the mean of the four measurements from the investigators versus Artificial Intelligence for regular lesions (top) and irregular lesions (bottom)

**Fig. 7:**
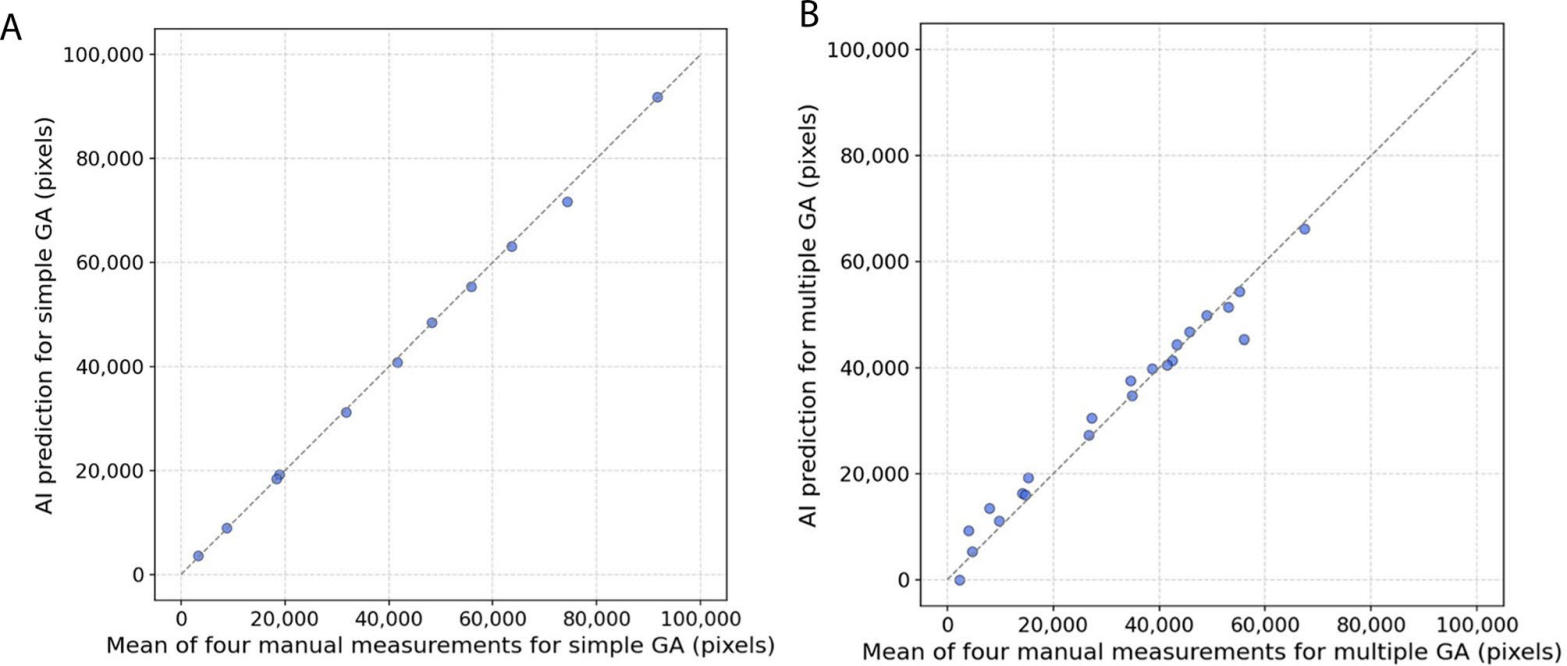
Scatter plot comparing the mean of the four measurements from the investigators versus Artificial Intelligence for simple lesions (top) and multiple lesions (bottom)

Agreement between the AI-based measurements and the mean of the four manual annotations was strong, with a Lin’s concordance correlation coefficient (CCC) of 0.992 (95% CI, 0.981–0.998) (Fig. 5). When stratified by lesion morphology, CCC values were 0.989 (95% CI, 0.816–0.997) for regular lesions and 0.988 (95% CI, 0.944–0.998) for irregular lesions (Fig. 6). Stratification by lesion number yielded CCC values of 0.999 (95% CI, 0.998–1.000) for single lesions and 0.984 (95% CI, 0.963–0.9954 for multiple lesions (Fig. 7).

**Fig. 8:**
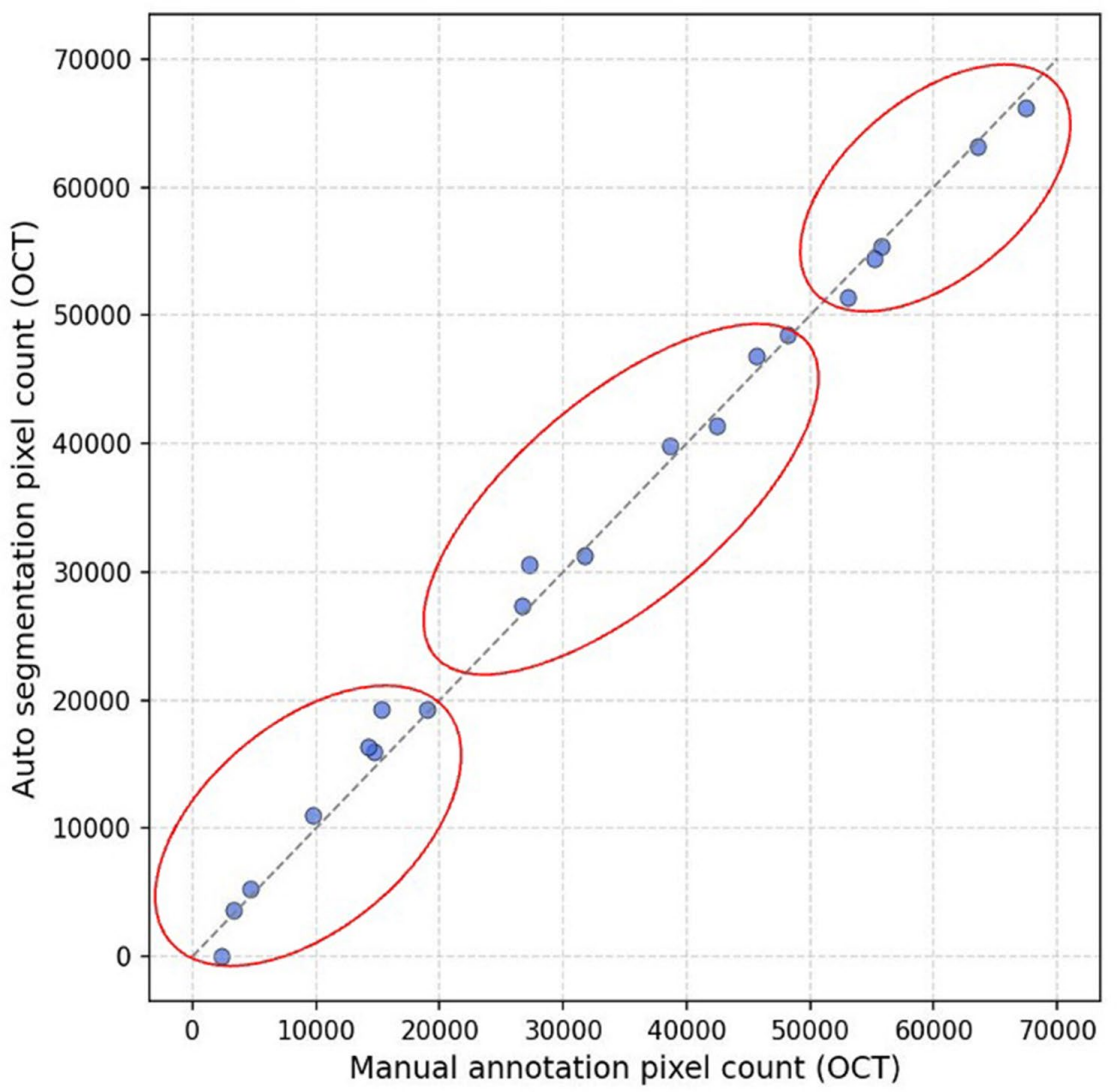
Scatter plot comparing the mean of the four measurements from the investigators versus Artificial Intelligence, stratified by lesion size: small (< 20,000 pixels), medium (20,000 to < 50,000 pixels), and large (> 50,000 pixels)

When analyzed according to lesion size, agreement was lower for small lesions (< 3.7 mm^2^; *n* = 12), with a CCC of 0.903 (95% CI, 0.742–0.974). Higher agreement was observed for medium-sized lesions (3.7–9.4 mm^2^; *n* = 13), with a CCC of 0.978 (95% CI, 0.940–0.994), and for large lesions (> 9.4 mm^2^; *n* = 8), with a CCC of 0.956 (95% CI, 0.676–0.998) (Fig. 8). Slightly lower agreement in small cases is explained by the potential interference of peripapillary atrophy in 2 cases of this group.

The AI execution time was 1.2 s for the initial case and 1.02 ± 0.14 s for the subsequent 20-eye dataset (Intel^®^ Core™ i9-14900, 2.00 GHz; 64 GB RAM), compared with 56.5 ± 17.3 min (range, 41–72 min) required for manual delineation of all 33 cases.

## Discussion

Historically, CFP has been used for the detection and follow-up of GA lesions because of its accessibility and ease of interpretation. However, delineation of GA on CFP is often challenging due to limited contrast and variability in GA presentation, particularly from a machine-learning perspective [1].

FAF imaging provides superior performance compared with CFP for measuring GA lesion area, but it has several limitations. Assessment of foveal involvement is difficult because of the intrinsically low FAF signal intensity in the foveal region. In addition, FAF does not allow evaluation of photoreceptor integrity and is susceptible to poor-quality images [8]. Semi-automated segmentation of GA area on FAF can be performed using the Region Finder software (Heidelberg Engineering) [17, 18]; however, manual correction is required, making the process subject to observer-dependent errors.

Absorption of short-wavelength light by the foveal pigment results in reduced FAF signal intensity at the fovea, complicating the assessment of foveal involvement [19]. This represents a significant limitation, not only in determining foveal involvement per se but also in assessing the distance between the GA lesion and the fovea, which is of major prognostic importance. Moreover, FAF is not universally available and requires specialized equipment, trained personnel, and specific camera settings to obtain high-quality images. For these reasons, optical coherence tomography (OCT) is considered more suitable for the evaluation of GA in routine clinical practice [8].

In contrast to two-dimensional FAF imaging, OCT provides three-dimensional visualization of retinal layers within the macular region. While FAF is limited to assessing retinal pigment epithelium (RPE) atrophy, OCT enables evaluation of changes at the level of the photoreceptor (PR) layers and the outer nuclear layer (ONL). Furthermore, OCT has become the standard imaging modality for the management of macular diseases and is widely available in clinical practice [20]. OCT examinations are patient-friendly, easy to acquire, and more reliable for assessing foveal involvement in GA lesions. OCT is also less affected by pupil size and media opacities, reducing the proportion of ungradable images.

Spectral-domain OCT (SD-OCT) devices offer an axial resolution of approximately 8 μm and are capable of detecting subtle early atrophic changes that may not be visible with other imaging modalities. Consequently, OCT may represent the most appropriate imaging modality for monitoring GA in routine clinical practice [8]. SS-OCT enables rapid image acquisition using an invisible scanning light source, allowing stable datasets to be obtained within approximately 1.5 s. In addition, SS-OCT provides relatively uniform image quality across B-scans, contributing to more consistent and reliable measurements. Moreover, even in eyes with early cataracts, the longer wavelength (~ 1 μm, compared to 840 nm in SD-OCT) employed by the DRI OCT Triton system allows improved visualization of the macula.

OCT imaging has also been used by an expert consensus group to classify GA into complete RPE and outer retinal atrophy (cRORA) and incomplete RPE and outer retinal atrophy (iRORA) [21]. More recent reports from the Classification of Atrophy Meetings (CAM) group have further refined and clarified this classification system [22–24].

Real-time assessment of GA and reliable manual quantification have become too labor-intensive to be feasible in routine clinical practice. Therefore, automated evaluation of GA secondary to age-related macular degeneration (AMD), including monitoring disease progression under treatment using artificial intelligence (AI), has become increasingly relevant for patient management and research [8].

Several authors have reported results on the automated detection of GA areas using AI-based approaches, employing different 2D and 3D systems, volumetric measurements, or B-scan–based analyses (Table 2) [8]. Most studies used Spectralis OCT devices and included sample sizes ranging from 18 to 192 eyes, reporting Dice similarity coefficients between 0.84 and 0.96 [25–30].

**Table 2:** Summary table of previously published studies

Authors	External Test Set	2D/3D	Imput	OCT Device	Method	Performance (mean DSC)
Mai et al. 2023 [25]	113 eyes/patients	3D	Volumen	Spectralis	3D to 2D	0.91
Pfau et al. 2020 [26]	25 eyes/patients	2D	“en-face” projection and layer thickness maps	Spectralis	Deep lab V3	0.96
Zhang et al. 2021 [27]	192 eyes of 110 patients	2D	B-scan	Spectralis	U-Net	0.91
Kalra et al. 2023 [28]	N/A	2D	“en-face“ projection and OCT B-scan	Spectralis and Cirrus	2 x U-Net	N/A
Derradji et al. 2021 [29]	18 volumetric OCTs	2D	B-scan	Spectralis	U-Net	0.84–0.88
Pramil et al. 2023 [30]	72 eyes of 51 patients	2D	“en-face “slab and sub-RPE slab	Plex Elite SS-OCT	U-Net	0.92

DSC: Dice Similarity Coefficient; RPE: Retinal Pigmentary Eptihelium;

From G.S. Reiter et al. Progress in Retinal and Eye Research 103(2024)101305

In our study, excellent levels of agreement were observed between the two investigators, with concordance values exceeding 0.99, supporting the reliability of the manual measurement system used as the reference standard. Agreement between manual measurements and AI-based quantification was also very high (> 0.98), confirming the accuracy of the proposed method with minimal time required for analysis. This performance supports its potential utility in routine clinical practice as well as in research settings. One key difference is that previous methods [7] operated on individual OCT B-scans, whereas the AI approach leverages projection images derived from the full OCT volume. By incorporating spatial context from neighboring pixels and adjacent structures, the AI can better capture the global extent and morphology of geographic atrophy, which improves detection accuracy and robustness. In addition, the AI model is based on SAM-2, a foundation segmentation model trained on over 600,000 annotated samples, enabling it to generalize well and produce more consistent and reliable segmentations compared with the earlier rule-based or slice-wise methods.

In this study, ground truth (GT) was generated from OCT *en façe* images, and the same dataset was used to train an AI model to extract GA areas. Therefore, we believe that both the GT delineations by the graders and the images estimated by the AI were well learned as reference data. When generating the AI output images, additional processing was applied to enhance boundary clarity.

In a recent study, Savastano et al. compared GA area measurements obtained using AI across multiple imaging modalities, including FAF, near-infrared (NIR) imaging, retromode (RM) imaging, and OCT angiography (OCTA), in 157 eyes. The intraclass correlation coefficient (ICC) between manual and automated measurements was 0.82 (95% CI, 0.78–0.84) for the FAF-based model, 0.81 (95% CI, 0.78–0.82) for the NIR model, 0.67 (95% CI, 0.64–0.71) for the RM model, and 0.77 (95% CI, 0.73–0.81) for the OCTA model. The authors concluded that automated performance was very good, although a slight overestimation of GA area was observed [31].

AI-based methods have also been reported for measuring RPE and ellipsoid zone (EZ) depletion areas on OCT images in 168 eyes from 110 patients with GA secondary to AMD who received at least three pegcetacoplan injections [32]. In a recent publication, Schmidt-Erfurth concluded that ophthalmologists may use AI-assisted OCT analysis to improve GA management by identifying patients most likely to benefit from treatment. AI-based analysis demonstrated that pegcetacoplan slowed EZ and RPE layer degeneration in patients with GA [33]. Recent studies have already incorporated AI-based GA measurements into research protocols [34, 35].

The main limitation of the present exploratory study is the absence of external validation. In the development of AI models for GA analysis, datasets are typically divided into a development set—including training and internal validation subsets—which were both performed in this study. Further studies with external validation using datasets from different samples to ensure the effectiveness of the AI software in diverse clinical environments will be needed to confirm these results. Small and irregular lesions yielded slightly worse results, although correlation figures exceed 0.9 in all cases. Another limitation is the relatively small number of eyes included; although comparable to some similar studies, a larger sample would indeed strengthen the conclusions reached.

The software is expected to be applicable to SD-OCT images, and further evaluation using SD-OCT datasets is planned. Given that GA assessment primarily relies on structural features that are consistently captured across OCT technologies, no significant differences between SS-OCT and SD-OCT images are anticipated to substantially impact the analysis. This will, however, be formally validated in future studies.

In this study, no manual corrections were applied to the results, and all outputs were presented exactly as generated by the AI in order to provide an objective evaluation of its performance. Nevertheless, we acknowledge the importance of flexibility in real-world clinical settings. For this reason, in practical implementations within commercial software, a manual editing function is typically available. In cases where the AI incorrectly delineates a lesion, the user can adjust the GA boundary directly on the en face image, after which the GA area and perimeter are automatically recalculated.

In conclusion, the accuracy of this new software for GA area measurement is substantial, demonstrating a high degree of repeatability and precision across different lesion types, while requiring minimal processing time.

## Data Availability

The datasets used and/or analysed during the current study are available from the corresponding author on reasonable request.
